# The healthy equine uterus harbors a distinct core microbiome plus a rich and diverse microbiome that varies with geographical location

**DOI:** 10.1038/s41598-022-18971-6

**Published:** 2022-08-30

**Authors:** G. R. Holyoak, H. U. Premathilake, C. C. Lyman, J. L. Sones, A. Gunn, X. Wieneke, U. DeSilva

**Affiliations:** 1 Department of Veterinary Clinical Sciences, Oklahoma State University, Stillwater, OK USA; 2 Department of Animal and Food Sciences, Oklahoma State University, Stillwater, OK USA; 3 College of Veterinary Medicine, Auburn University, Auburn, AL USA; 4 School of Veterinary Medicine, Louisiana State University, Baton Rouge, LA USA; 5 School of Agricultural, Environmental and Veterinary Sciences and Gulbali Institute, Charles Sturt University, Wagga Wagga, NSW Australia; 6 Present Address: Department of Pathology and Laboratory Medicine; Center for Genomics, Anne and Robert H. Lurie Children′s Hospital, Northwestern Feinberg School of Medicine, Chicago, IL USA

**Keywords:** Agricultural genetics, Microbial genetics

## Abstract

The goal of this study was to understand the composition and existence of the resident uterine microbiome in healthy mares and to establish the presence of a core microbiome for the healthy equine uterus. We analyzed the microbiomes of 35 healthy mares that are long-time residents of three farms in Oklahoma, Louisiana, and Australia as well as that of 19 mares purchased from scattered owners in the Southern Mid-Western states of the United States. Over 6 million paired-end reads of the V4 region of the 16S rRNA gene were obtained resulting in 19,542 unique Amplicon Sequence Variants (ASVs). ASVs were assigned to 17 known phyla and 213 known genera. Most abundant genera across all animals were *Pseudomonas* (27%) followed by *Lonsdalea* (8%), *Lactobacillus* (7.5%), *Escherichia/Shigella* (4.5%), and *Prevotella* (3%). Oklahoma and Louisiana samples were dominated by *Pseudomonas* (75%). *Lonsdalea* (28%) was the most abundant genus in the Australian samples but was not found in any other region. Microbial diversity, richness, and evenness of the equine uterine microbiome is largely dependent on the geographical location of the animal. However, we observed a core uterine microbiome consisting of *Lactobacillus, Escherichia/Shigella, Streptococcus, Blautia, Staphylococcus, Klebsiella, Acinetobacter,* and *Peptoanaerobacter*.

## Introduction

For decades the standard dogma was that the uterus was maintained as a sterile environment despite the vagina being colonized with commensal bacteria^1^. Even though culture-based studies laid out the foundation of our understanding of the reproductive tract microbiota as far back as three decades ago, there was an indication that culture-based methodologies in aiming to identifying microbial populations may underestimate diversity and overestimate the role of culturable bacteria^2^. Focusing on culturable bacteria, often the minority members of microbial communities, enhances the risk of missing and therapeutically disrupting those microbes that are more abundant^3–5^. Furthermore, the general consensus now is that culture-based technologies detects less than 10% of the resident microbial community in a sample^6^.

With the advent of 16S rRNA gene-based bacterial detection and identification techniques, the sterile womb paradigm has been challenged through fluorescence in situ hybridization (FISH) with 16S rRNA targeted probes in pregnant and non-pregnant women^7^. Different microbial communities with variable richness and diversity have been found among other species in distinct areas of the reproductive tract^8^. A recent study on the resident microbiota of the canine vagina (cranial) and endometrium revealed that, they both are home to a diverse microbiome; more specifically, a distinct dissimilarity exists in the structure and diversity of the endometrial microbiome in comparison to the microbiome of the cranial vagina^9^. During another study based on 16S rRNA gene amplicon sequencing of bovine, ovine, and porcine vaginal microbiomes were found to be distinctly different from how they were described using culture-based techniques^10^. Therefore, it is clear that the traditional culture-based techniques lack in the ability to capture the inherent diversity of the native microbial ecosystems that prevail within the mammalian reproductive tract.

The clinical literature continues to describe the uterus of a normal (healthy) mare as a site that does not host a resident microbial community^11^. However, previous studies based on in vitro culturing indicate that, a variety of microorganisms may be present in the uterus of a clinically healthy mare^12^. However, failure to remove pathogenic bacteria, sperm and other inflammatory substances may cause post breeding endometritis in mares^13^. Furthermore, several bacterial genera such as *Streptococcus*, *Escherichia* and *Staphylococcus*, working as opportunistic pathogens in the equine uterus, are known to cause pregnancy loss and infertility in mares due to infectious bacterial endometritis^14,15^.

The importance of having a comprehensive understanding of the resident microbiota of the reproductive tract of a healthy mare, or any domestic animal species, lies in the need to understand the role played by these commensal microorganisms and how dysbiosis may impact the overall health of the animal. Furthermore, decoding the composition of the uterine microbiome plays a vital role in comprehending the effect of broad spectrum antibiotics on shifting the overall composition of the resident microbiota possibly playing important roles in the reproductive process and fertility^16^.

We began our metagenomics investigations in the mare to document the underestimation of microbial diversity with common culture methods relative to the presence of microbial DNA using the approach over 10 years ago^17^. Subsequently, Schnobrich et al.^18^ described Next Generation Sequencing (NGS), culture, and cytology results from 29 mares with suspected endometritis. The main problem with these analyses were the dependence on culture and sequencing depth.

In this study, we attempted to comprehensively analyze the resident microbiome of 54 clinically healthy mares from four different geographical locations in two continents and attempted to establish a stringent core microbiome of the healthy equine uterus. To the best of our knowledge, this is the first extensive study based on 16S rRNA gene amplicon sequencing, conducted to gain a comprehensive understanding about the uterine microbiome of the healthy mare.

## Results

### Sequencing results and data analysis statistics

Sequence analysis of 16S rDNA amplicons resulted in 6,486,441 paired-end reads from 56 samples at 115,829 ± 47,952 reads per sample ranging from 32,222 to 147,965. After removal of low-quality and ambiguous reads, 5,868,329 sequence reads were retained for downstream analysis. From these sequence reads, a further 3,784,490 was retained after sequence variant inferring, sequence read merging and chimera removal, which gave rise to 19,776 unique amplicon sequence variants. About 96% of the inferred sequence variants were retained during chimera removal, thus indicating that initial data cleansing steps were successful. Subsampling based on least number sequences for a sample was performed on all samples to normalize data and remove sequence bias before any of the downstream processing.

**Figure 1:**
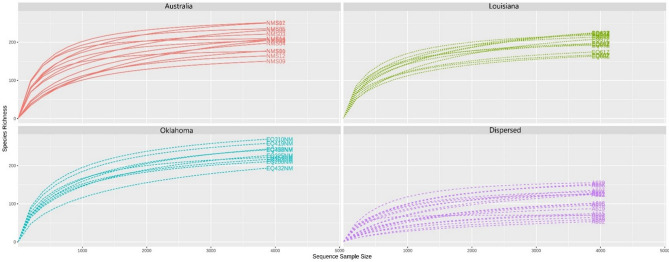
Rarefaction curves for all samples used for this study. Each curve is color coded based on the group it belongs to; Australia (n = 14), Oklahoma (n = 9), Louisiana (n = 12) and Dispersed (n = 19).

A taxonomy rarefaction curve indicated that the sequencing depth used in this study was sufficient to saturate species richness in all samples (Fig. 1).

### Alpha diversity

**Figure 2:**
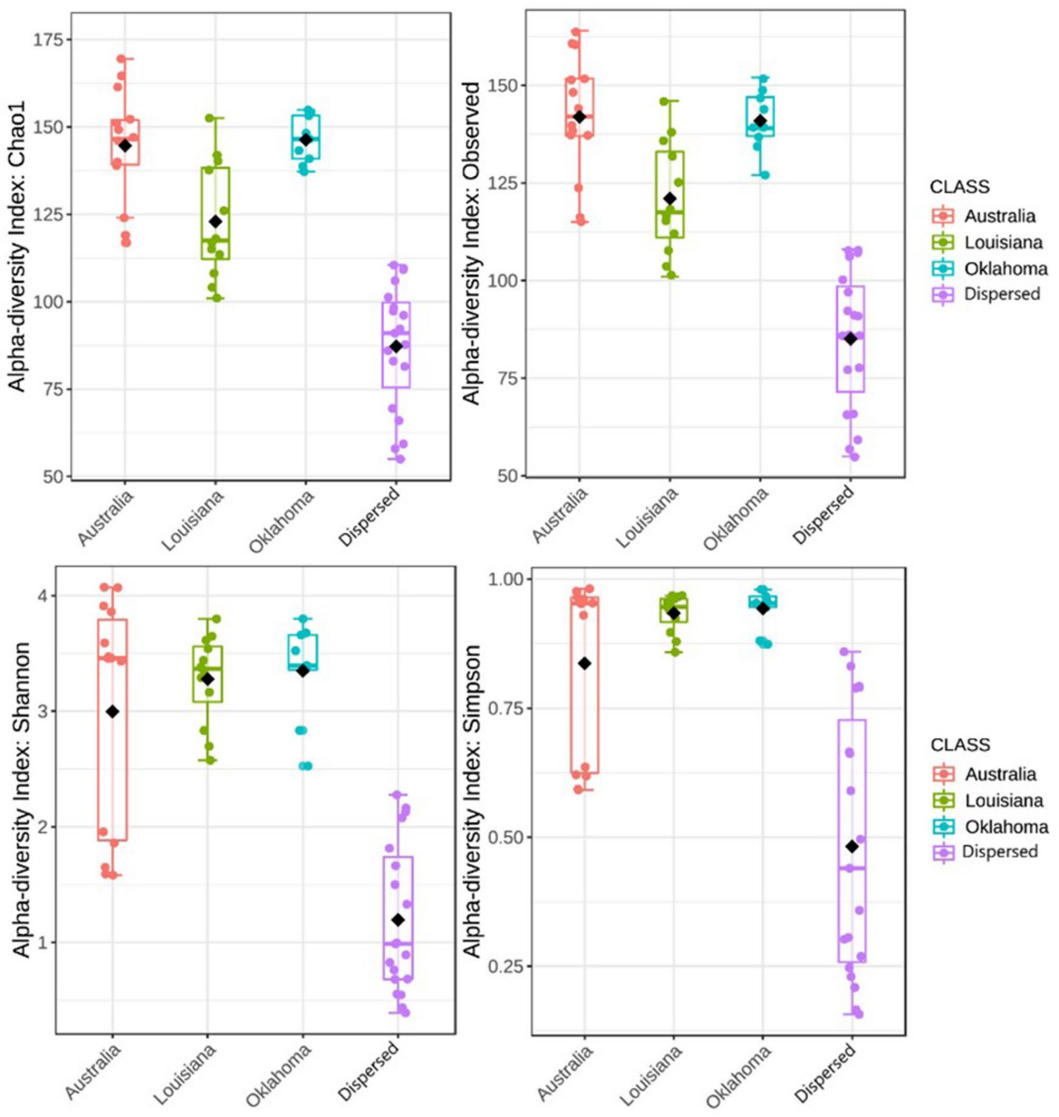
Box plot representing the alpha-diversity values for microbial communities recovered from endometrial lavage samples of healthy mares. The samples are grouped, and color coded according to their geographical origin (i.e., Oklahoma, Louisiana, Australia and Dispersed). Chao and Observed ASV metrices measure species richness while Shannon and Simpson indices measure evenness. ANOVA (Chao1 and Observed ASVs) and Kruskal–Wallis tests (Shannon and Simpson) were utilized to conduct between group comparisons.

Alpha diversity estimates for species richness and evenness were calculated for geographically grouped samples. Chao1 and Observed ASV metrices were used to calculate species richness, while Shannon and Simpsons Indices were used to calculate evenness^19^. Between group comparisons for Chao1 and Observed ASV metrices were performed using ANOVA, while Kruskal–Wallis test was used for Shannon and Simpson indices^20^. All four alpha diversity measures indicated that the uterine microbiomes of mares from four different geographical regions were significantly different in species richness and evenness (p < 0.05) (Fig. 2, Table 1). Australian samples were the highest in species richness, while Oklahoma samples were leading in species evenness.

**Table 1:** Between group comparison statistics for alpha diversity measures of species richness (Observed ASVs and Chao1) and evenness (Shannon and Simpson) for endometrial lavage samples from Oklahoma, Louisiana, Australia and Dispersed.

Alpha Diversity Index	Statistical test	Test statistic	p-value
Shannon	Kruskal–Wallis	31.425	6.9169e−07
Simpson	Kruskal–Wallis	31.62	6.2927e−07
Observed ASV	ANOVA	61.378	8.8234e−17
Chao1	ANOVA	54.163	9.8414e−16

To further ascertain the source of this intergroup difference in alpha diversity, pair-wise comparisons were conducted using Tukeys HSD and Dunn Test with B–H correction^21^ on species richness and evenness metrices respectively (Table 2). The pair-wise comparisons between the Dispersed group and all other groups were significantly different (p-adj < 0.05) in both species’ richness and evenness. Pairwise comparisons between Oklahoma and Australia deemed not significant for both species richness and evenness (p-adj > 0.05), while both Louisiana–Australia and Oklahoma–Louisiana comparisons revealed to be statistically significant in terms of species richness (p-adj < 0.05).

**Table 2:** Adjusted p-values for the pairwise comparisons of species richness and evenness metrices for healthy mare endometrial lavage samples, based on their geographical origin. Observed ASVs and Chao1 column contain post-hoc adjusted p-values derived using Tukeys HSD. Shannon and Simpsons columns contain the same derived using Dunn Test with B–H correction^21^. Cells containing significant adjusted p-values are colored.

Pair-wise comparison	Adjusted p-value for each alpha diversity index/metric			
	Observed ASVs	Chao1	Shannon	Simpsons
Louisiana–Australia	2.15E^−05^	5.73E^−05^	9.88E^−01^	5.03E^−01^
Oklahoma–Australia	2.73E^−01^	2.62E^−01^	1.00	5.71E^−01^
Dispersed-Australia	0.00	0.00	6.90E^−05^	1.97E^−04^
Oklahoma–Louisiana	2.97E^−02^	5.84E^−02^	8.41E^−01^	8.98E^−01^
Dispersed-Louisiana	2.00E^−07^	7.00E^−07^	5.18E^−05^	2.77E^−05^
Dispersed-Oklahoma	0.00	0.00E	6.35E^−05^	4.79E^−05^

### Beta diversity

**Figure 3:**
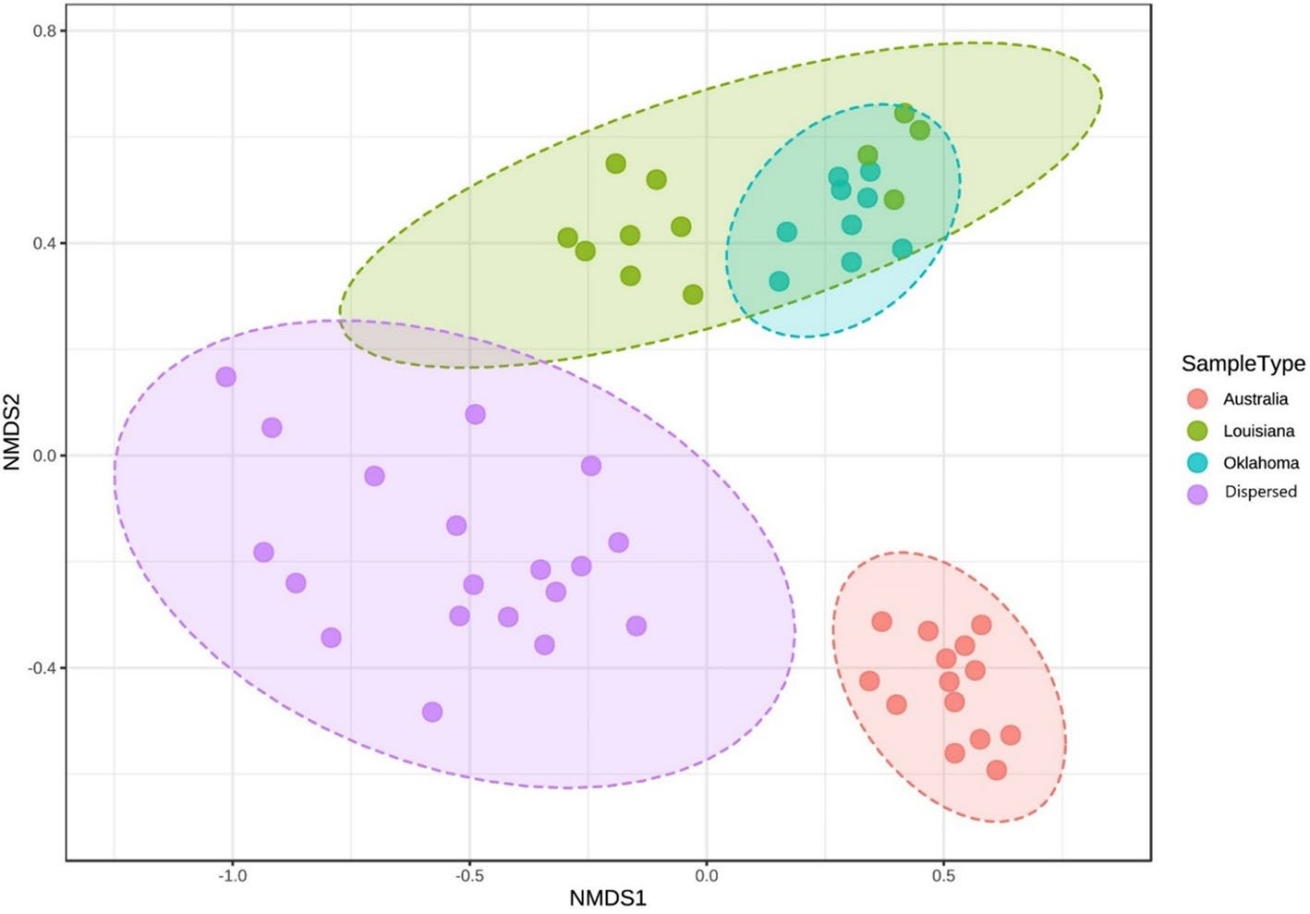
Non-metric multidimensional scaling (NMDS) plots depicting the distribution of endometrial lavage samples based on their microbial composition (beta diversity). The samples are color coded based on their geographical origin. NMDS1 and NMDS2 values were calculated based on the Bray–Curtis Index^23^. The conventional 95% confidence interval around the centroid of each grouping (based on its multivariate t-value distribution) is marked by the ellipses^22^.

Beta diversity values for all samples were calculated at genus level using the Bray–Curtis dissimilarity matrix and plotted using NMDS (Non-metric Multidimensional Scaling) ordination method available in MicrobiomeAnalyst^22^. Samples formed clear clusters based on their geographic origin (Fig. 3). Those originating from Oklahoma and Louisiana formed close, overlapping but distinct clusters, whereas samples from the Dispersed group and Australian animals clustered clearly apart. Permutational Multivariate Analysis of Variance (PERMANOVA) on the beta diversity values^23^ revealed that the distinct clustering visible on NMDS plots are statistically significant (F-value: 23.894; R-squared: 0.5891; p-value < 0.001), thus confirming that there is a significant difference in the composition or the microbial community structure recovered from endometrial lavage samples of animals from the Dispersed group, Oklahoma, Louisiana and Australia.

### Hierarchical clustering

**Figure 4:**
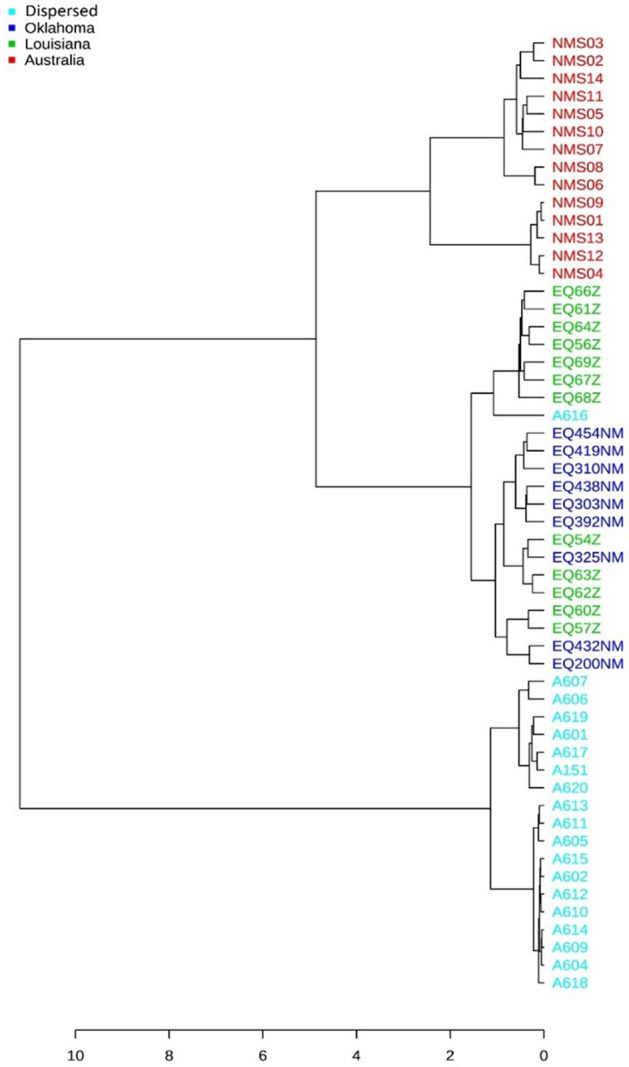
Hierarchical clustering dendrogram drawn at genus level, using Bray–Curtis Index as the distance measure and Ward’s method as the clustering algorithm^24^. Samples from Australia, Oklahoma, Louisiana, and Dispersed animals are color coded based on their geographical origin.

A dendrogram depicting the hierarchical clustering of the samples was drawn at genus level using Bray–Curtis index as the distance measure and Ward method as the clustering algorithm^24^ (Fig. 4).

Similar to the beta-diversity plots, three clear clusters were observed in the dendrogram as well. Australian and Dispersed samples (other than sample A616) formed clear separate clusters far apart, while the Oklahoma and Louisiana samples grouped together to form most of the third cluster. However, the separation between Louisiana and Oklahoma groups was less apparent when compared to the beta diversity plots.

### Microbial community structure

Sequence analysis of 16S rDNA amplicons resulted in 6,486,441 paired-end reads. Subsequent removal of low-quality, ambiguous, and non-bacterial sequences resulted in 3,716,157 sequence reads. These resulted in 19,542 unique amplicon sequence variants (ASVs) that were used to study the composition of the equine endometrial microbiome. These 19,542 ASVs were assigned to 17 known phyla (Supplementary Table S1) and 213 known genera (Supplementary Table S2) by DADA2. Proteobacteria (~ 48%) was the most abundant phylum, followed by Firmicutes (30%), Bacteroidetes (12%), Actinobacteria (5%), Tenericutes (2%), and Kiritimatiellaeota (1%). These 6 phyla accounted for 98% of total abundance. Proteobacteria, Firmicutes, Bacteroidetes and Actinobacteria and Kiritimatiellaeota were observed across all samples. Tenericutes were only observed in samples from Oklahoma and Louisiana. Firmicutes were the most abundant phylum (52%) in Louisiana samples, while both Proteobacteria (36%) and Firmicutes (36%) emerged as the top two equally abundant phyla in Oklahoma. Following the overall trend, samples from both Australia (40%) and Dispersed (80%) were dominated by Proteobacteria.

At genus level, *Pseudomonas* emerged as the most abundant (27%) genus cumulative across all samples. The rest of the top 5 genera were *Lonsdalea* (8%), *Lactobacillus* (7.5%), *Escherichia/Shigella* (4.5%) and *Prevotella_9* (3%), amounting to 51% of the total abundance. The most abundant genus in Australian samples was *Lonsdalea* (28%). Interestingly, *Lonsdalea* was not detected in any of the other samples. Samples from Oklahoma and Louisiana were dominated by *Lactobacillus* (14%) and *Escherichia/Shigella* (13%). These samples also had higher species diversity than Australian or Diverse groups. Out of the 19,542 ASVs analyzed, 9334 ASVs were not assigned a taxonomic classification at genus level. However, the vast majority of these had a taxonomic classification at Phylum level or below, only 371 ASVs remained unclassified at phylum level.

### The Core-microbiome

The core microbiome was defined as genera present in all samples at an abundance of at least 0.1%^25^. Eligible genera from each group were overlapped using a Venn diagram^26^. A collection of eight genera consisting of *Lactobacillus, Escherichia/Shigella, Streptococcus, Blautia, Staphylococcus, Klebsiella, Acinetobacter,* and *Peptoanaerobacter* were present in all samples and was designated as the core microbiome of healthy equine uterus.

### Phylogeny of the normal mare microbiome

**Figure 5:**
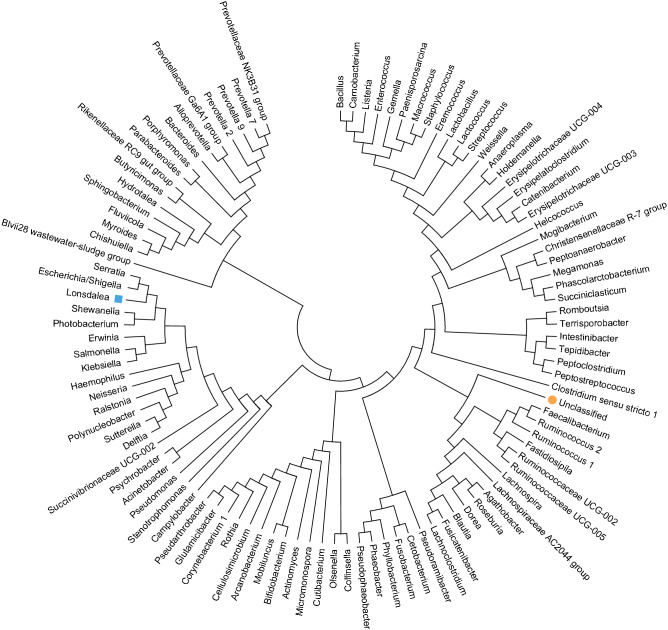
Molecular Phylogenetic analysis of the endometrial microbial community recovered (represented by the most abundant 104 ASVs). The Maximum Likelihood method, based on Tamua-Nei model, was used to infer the evolutionary relationships. A set of probable tree topologies were inferred from a Maximum Composite Likelihood (MCL) pairwise distance matrix, using Neighbor-Join and BioNJ algorithms. The illustrated tree is the one with the highest log likelihood. All pertaining analyses were conducted using MEGA7^65^.

Out of the 104 ASVs used for this analysis, one, was not assigned with a classification at genus level but belonged to order Clostridiales. In the phylogenetic tree illustrated in Fig. 5, this was observed to correctly cluster alongside other Clostridiales. Lonsdalea, previously unknown to inhabit the equine reproductive tract, and the most abundant genus in the Australian samples, was observed to accurately cluster alongside *Escherichia/Shigella* which share the same taxonomic family. This further confirms the accuracy of assignment to amplicon sequence variants and their subsequent taxonomic classification.

### Differentially abundant taxa

LEfSe or linear discriminant analysis (LDA) effect size, an algorithm for class comparison of high dimensional data^27^ and DESeq2, a method utilized for differential analysis of count data^28^, both implemented in Microbiome Analyst was used to identify differentially abundant taxa, or biomarkers, based on the geographic origin of the endometrial lavage samples. DESeq2 was able to identify 92 significant features based on false discovery rate (FDR < 0.05), and LEfSe identified 194 (adjusted FDR < 0.05, LDA > 2.0) significant features.

**Figure 6:**
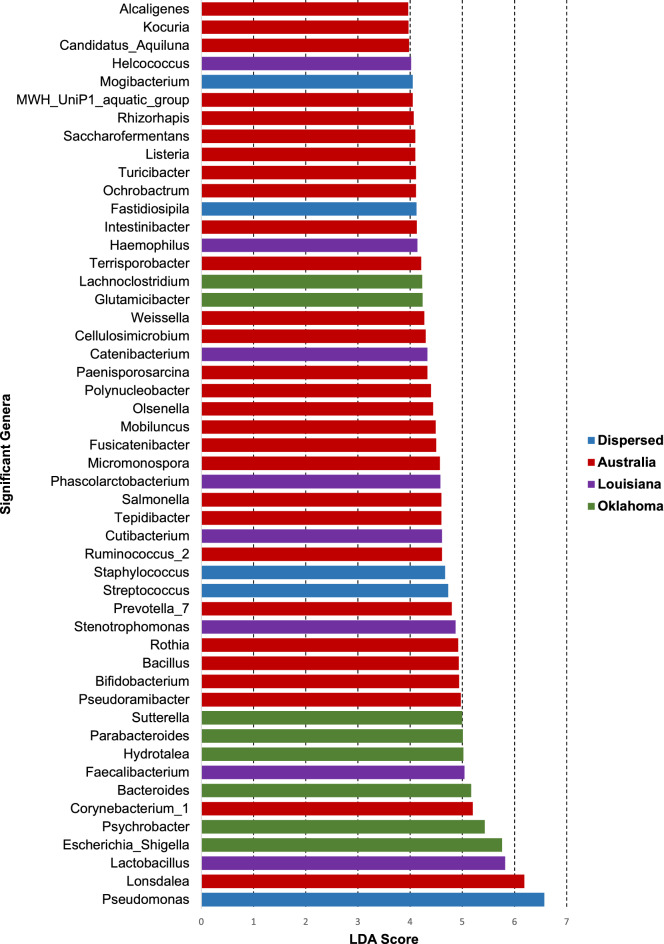
Significant features or bio markers that are differentially abundant in the endometrial lavage samples when grouped according to their geographical origin. 92 consensus differentially abundant genera were identified using LEfSe and DESeq2 algorithms, the top 50 of them are illustrated in the figure in ascending order of their LDA score. The bars, representing individual genera are color coded to indicate the sample group (geographical origin) in which they are most abundant.

A consensus list of 92 significant features was arrived upon (Supplementary Table S3) based on the overlapping features from the two independent lists derived above^19^. *Pseudomonas, Lonsdalea, Lactobacillus, Escherichia/Shigella,* and *Psychrobacter* emerged as the top 5 biomarkers with a LDA score > 5.0. *Pseudomonas, Lonsdalea,* and *Lactobacillus* were significant in the Dispersed, Australian and Louisiana samples respectively, while both *Escherichia/Shigella,* and *Psychrobacter* appeared to be significant in Oklahoma samples (Fig. 6).

## Discussion

The mammalian uterus has long been considered a sterile environment to sustain life^1^. However, this paradigm was challenged via some seminal studies in humans^7,29^. Since that time, the presence of a rich and dynamic microbiome in the mammalian endometrium was established in several species^9,23,30–33^. We and others have previously established that the uteri of clinically healthy mares host a resident microbiome^12,17,34^. The importance in understanding this potential shift in accepted dogma is underscored by the fact that many pregnancy related complications in humans^35^, and other mammals, especially those in important livestock species^36,37^, are well known to be of bacterial origin. Equine endometritis occurs in 25–60% of breeding mares^38,39^ resulting in infertility, postpartum metritis as well as septicemia and fatality in newborn foals^40^. Historically, genera such as *Staphylococcus, Escherichia, Pseudomonas,* and *Klebsiella* were considered to be invasive pathogens indicating dysbiosis in mares^41^. However, this and our previous studies indicate that these four genera are common members of healthy mare uterine microbiome underscoring the importance of understanding the structure and diversity of healthy uterine microbiome of the mare and to define a “core microbiome”.

The alpha and beta diversity of bacterial communities recovered from equine uteri in this study indicate that the species diversity, richness and evenness of the healthy mare uterine microbiome defer significantly based on the geographical origin of the animal (Fig. 2, Table 1). The pairwise comparisons of alpha diversity indices indicate that microbiome of animals listed as Dispersed is significantly different in species richness and evenness from those of Oklahoma, Louisiana and Australian animals.

Similarly, beta diversity analyses using NMDS plots (Fig. 3) show that Oklahoma and Louisiana samples cluster together and the Dispersed group, as expected, forms a separate cluster with increased species diversity. The Australian samples, that were farthest away geographically, formed a tight cluster away from the other three groups. The clustering makes sense as Oklahoma, Louisiana, and Australian animals were long-term residents of respective university farms and were expected to cluster together. Dispersed animals were purchased from an Oklahoma horse purveyor who collects animals from a dispersed area in Southern Midwestern states of United States. As expected, they were the most diverse, yet they were closer to US samples than Australian samples. Australian animals clustered together away from other animals. Hierarchical clustering (Fig. 4) also depicts three clear clusters with Oklahoma and Louisiana samples clustering together, dispersed samples mostly clustering on their own and the Australian samples forming their own distinct cluster.

We generated 6,486,441 paired-end sequences in this study that yielded 19,542 unique ASVs. They were assigned to 17 known phyla and 213 known genera. Consistent with other mammalian uterine microbiomes^9,23^, the equine uterus was dominated by Proteobacteria (48%), Firmicutes (30%), and Bacteroidetes (12%). As expected, there were geographical differences in the phyla present in samples. Tenericutes were only observed in horses from the university farms in Oklahoma and Louisiana. They were not observed in dispersed animals, many of whom would have been from Oklahoma. This is surprising due to the fact that they were one of the top 6 phyla in the study, but their inconsistency in reproductive tract metagenomes have been observed before^42^.

**Figure 7:**
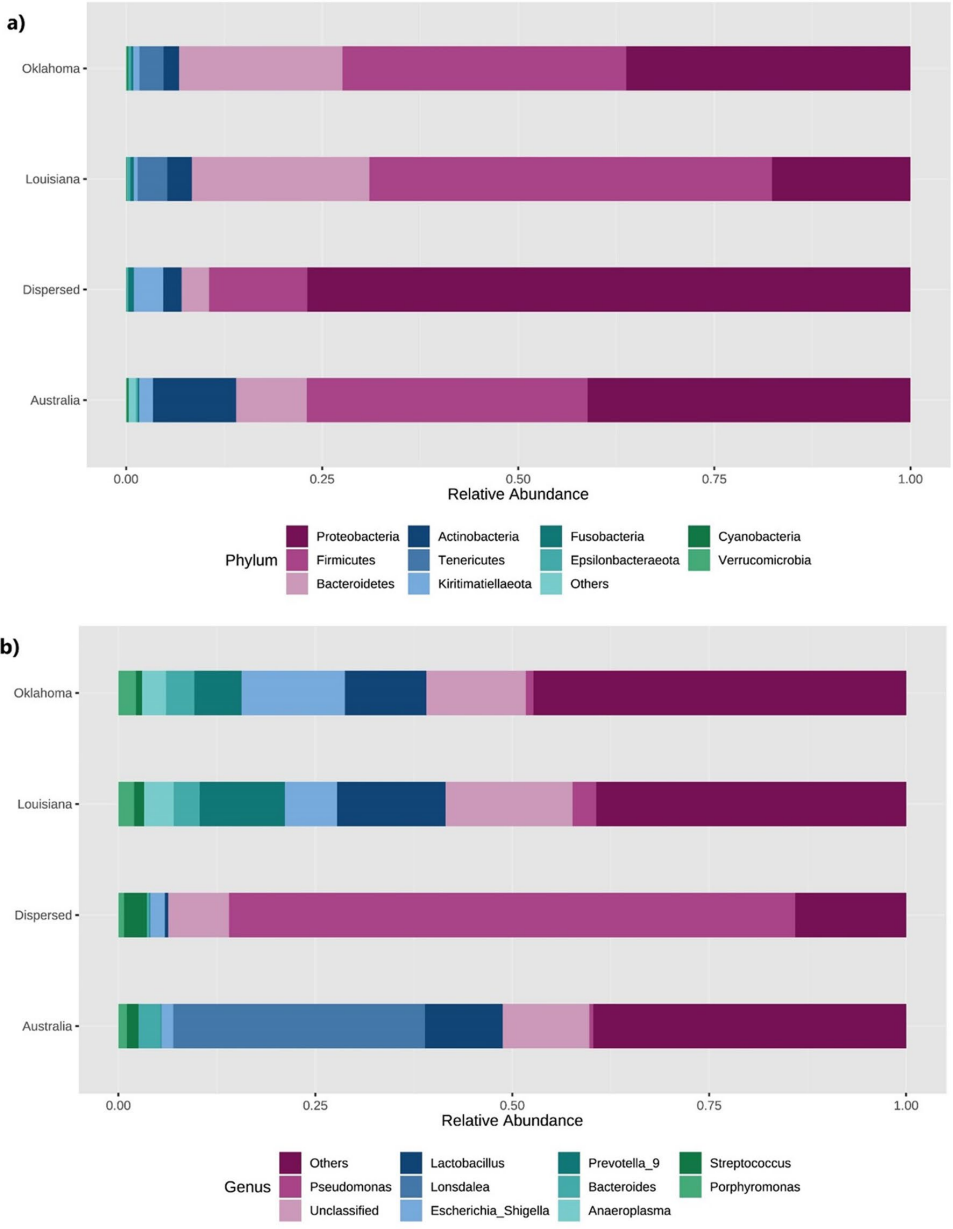
The microbial community structure and composition of endometrial lavage samples of mares belonging to Oklahoma, Louisiana, Australia, and Dispersed groups**.** Stacked bar graphs generated depict the relative abundance of the top 10 phyla (**a**) and genera (**b**) identified and grouped according to their geographical origin.

At the genus level *Pseudomonas* was the most abundant genus (27%). This is consistent with our previous work where we identified *Pseudomonas* as the most abundant genus in the canine endometrium as well^9^. The prevalence of *Pseudomonas* in the present study was largely driven by it dominating the uteri of animals from the Dispersed group (Fig. 7). One could speculate the presence to differences in management of those animals prior to our acquisition. *Pseudomonas aeruginosa* is thought to be an opportunistic pathogen that is responsible for many instances of equine endometritis^43,44^. Indirect evidence suggests that organisms belonging to the genus *Pseudomonas* in our study consists of several species. We are unable to reliably assign species with the SILVA database we used for taxonomic classification^45^. However, we are in the process of isolating and fully characterizing the *Pseudomonas* spp. identified in equine uteri.

Species composition of Australian samples revealed an interesting trend. The dominant genus in many samples were *Lonsdalea.* In 5/14 samples *Lonsdalea* represented over 60% of the organisms identified. In another two samples they exceeded 20%. This was an intriguing finding as *Lonsdalea* has not previously been reported in mammals. It is a relatively new bacterial genus that previously belonged to the genus *Brenneria*. *Lonsdalea* (as well as *Brenneria*) is a known phytopathogen that belong to Enterobacteriaceae family and is a pathogen of poplar and oak trees^46^. *Lonsdalea quercina* has been isolated from the surfaces of flight membranes of lesser horseshoe bats in the Czech Republic^47^. *Lonsdalea* was not detected at all in any of the other locations. However, organisms belonging to the genus *Erwinia* was identified in 5 animals from the Oklahoma group and one animal from the Louisiana group with a groupwise relative abundance of 0.94%. This is interesting as *Lonsdalea* and *Brenneria* were formerly classified as *Erwinia* prior to recent taxonomic reclassification^47^. *Erwinia* and *Brenneria* were also isolated from the gut microbiome of redbanded stink bugs (*Piezodorus guildinii*)^48^*.* We considered the possibility of *Lonsdalea* being an environmental contaminant in the Australian samples. However, negative controls including those for lavage medium and equipment failed to detect any *Lonsdalea.* The large contribution of this one genus to Australian samples, and its presence in majority of the animals tested is intriguing and rules out a transient environmental infection. As our Australian samples came from one semi-closed herd in one location, further investigation is required before the significance of *Lonsdalea* in equine uteri could be determined. It is also possible that *Lonsdalea* found in Australia and *Erwinia* found in Oklahoma and Louisiana belong to a hitherto unclassified organism(s) belonging to the same superfamily.

**Figure 8:**
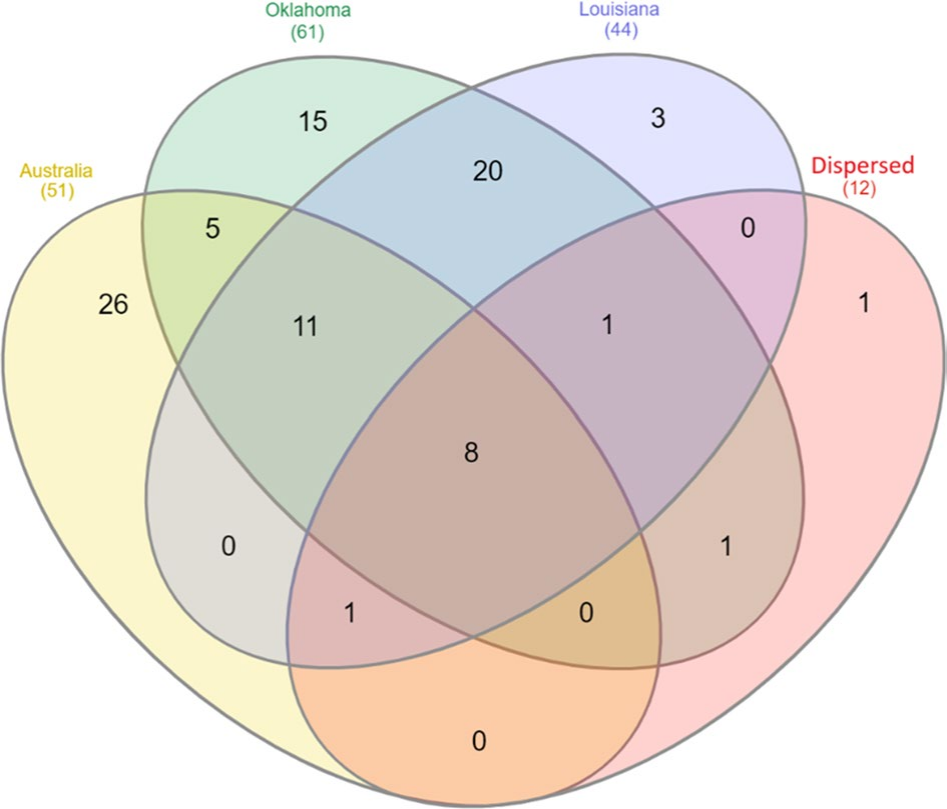
A four set Venn diagram illustrating the most prevalent bacterial genera identified in endometrial lavage samples from Australia, Oklahoma, Louisiana, and Dispersed animals (with a groupwise relative abundance > 0.1%). Eight bacterial genera (*Lactobacillus, Escherichia/Shigella, Streptococcus, Blautia, Staphylococcus, Klebsiella, Acinetobacter, Peptoanaerobacter*) in the intersection of all four sets, was defined as the core microbiome.

One of our major goals in this study was to identify and establish a core microbiome for the healthy mare uterus. We used a comparatively strict criterion of a groupwise prevalence of 100% and a 0.1% groupwise abundance^25^. We identified a core microbiome constituting of the following 8 genera in the mare endometrium. They were *Lactobacillus, Escherichia/Shigella, Streptococcus, Blautia, Staphylococcus, Klebsiella, Acinetobacter,* and *Peptoanaerobacter* (Fig. 8).

*Lactobacillus* (7.5%) had the third highest relative abundance across all samples (after *Pseudomonas* and *Lonsdalea*). *Lactobacillus* has long been observed as a dominant commensal organism in the human vagina and is known to play a role in gestational length, parturition and maintaining a healthy vaginal microbiome^49^. Furthermore, *Lactobacilli* are reported as a dominant member of the human uterine microbiome as well with implications in implantation success^50,51^. Previous studies in the mare reported that the presence of *Lactobacillus* in the equine uterus was low compared to humans and was reported at < 2% in abundance^52^. In this study, we found *Lactobacillus* to be the most prevalent genus in the core microbiome with an abundance of 7.5%. *Escherichia/Shigella, Streptococcus* and *Klebsiella* have previously been reported from uterine lavages of mares suspected of endometritis in a previous study^18^. *Blautia* and *Acinetobacter* have not been reported from the equine uterus to date, however, they have been reported as abundant in the human uterus^50^ and in the human gut microbiome^53^. *Peptoanaerobacter* has not been reported from reproductive tracts before but has been identified associated with human periodontal diseases^54^.

We were cognizant of the debate surrounding the placental microbiome and the role of reagent contamination^55,56^. Unlike the placenta, the uterine microbiome has a reasonable biomass. However, we collected negative controls for equipment and reagents at each stage of analysis to account for any reagent contamination and removed all detected sequences from further analysis^57^.

From this study, we conclude that the healthy equine uterus harbors a rich and diverse microbiome. We have illustrated the structure and composition of this microbiome with detailed analysis of endometrial microbiomes obtained from animals from four distinct geographical regions in two continents. We acknowledge that differences in climate and feed could have contributed to some of the variations we have identified. We bridged a research gap that existed on the commensal uterine microbiome of a healthy mare. The total composition of the microbiome varies with the geographical region while still maintaining a distinct core microbiome across all animals tested. We do realize that only a limited number of geographical locations were assayed in this study. We have established a platform for future research on elucidating the uterine microbiome at species level using full-length 16S rDNA sequencing and establishing the functional attributes of the microbiome utilizing whole metagenome sequencing^58–60^. We hope that other researchers would build on this study and further refine the healthy equine uterine microbiome. Furthermore, this paves the way for developing microbes as pre- or probiotics to enhance and maintain the natural balance of the uterine microbiome, thus improving the overall reproductive health of the mare.

## Methods

### Animals

Fifty-four geographically dispersed and clinically mature healthy mares between 4 to 18 years of age were used for the study. Out of the 54 animals, 9, 12, and 14 were located on university farms located in Stillwater, Oklahoma, Baton Rouge, Louisiana, and Wagga Wagga, NSW, Australia, respectively. These mares were long-term residents of their respective farms. They are labeled Oklahoma, Louisiana, and Australia in this study. The remaining 19 mares were purchased from an Oklahoma horse purveyor who is a source to regional veterinary practices for embryo recipient mares and collects animals from a wide multi-state area in the Southern Midwestern states of the US. They are labeled “Dispersed” in the study. These mares were quarantined for 4 weeks upon arrival at the Oklahoma State University farm and were allowed to acclimatize on pasture for 2 + months before samples were collected. Dispersed mares and Oklahoma mares shared the same facility and identical management conditions for multiple months although they never co-mingled in the same pasture. All mares used in the study were anatomically and clinically normal with no history of reproductive problems. The mares were given a complete pre-study breeding soundness exam so that the study included only historically reproductively normal mares with no clinical evidence of endometritis. Mares used in this study were of different breeds. All animal studies were approved by the Institutional Animal Care and Use Committees of Oklahoma State University (VM-17-25 and IACUC 20-50), Louisiana State University (#17-046), and Charles Sturt University (A18086). This study is reported here in accordance with ARRIVE guidelines^61^.

### Small volume lavage (SVL)

Following a thorough cleaning of the perineum and external genitalia, uterine luminal endometrial samples were collected using saline infused through an aseptic protected double-guarded system. To avoid contact with the vestibule and cranial vagina the first protective sleeve containing a double-guarded system was carried to the external os of the cervix where it was brought into contact with the cervix. A second tube (36 Fr. uterine lavage catheter) containing the sample catheter was inserted well into the cervix and the sample catheter (stallion urethral catheter) was then introduced deep into body of the uterus. 150 ml of saline was infused through the sample catheter, allowed 30 s of contact within the lumen prior to efflux collection via gravity flow and or aspiration. The initial 50 ml were not saved to ensure the sample collected had been within the uterine lumen. The samples were immediately placed on ice and transported to the laboratory for processing. This technique was proven to be sufficient for uterine microbial sampling in previous studies^62,63^. Control samples consisting of the saline flushed through the protected double-guarded system, including the same lubricants used for sample collection from both the US and Australia were collected and analyzed alongside the uterine samples. SVL samples from Louisiana and Australia were shipped to Oklahoma State University for further analysis.

### DNA isolation

All DNA extractions used in this study were performed in the same laboratory by a single investigator using identical reagents and laboratory conditions. Fifty mL of SVL obtained for each sample was spun down at 4000 rpm for 30 min at 4 °C. The pellet was then re-suspended in 1.5 ml of the same solution. Two hundred fifty µl of the resuspension was used for total DNA extraction using QiAamp DNA mini kit^®^ (Qiagen, Germantown, MD) based on manufacturer’s instructions. DNA quality and quantity was measured using a NanoDrop^®^ ND-1000 spectrophotometer. ZymoBIOMICS^®^ Microbial Community Standard (Zymobiomics, Irvine, CA Cat. D6300) containing a known composition of microbiota was used as the positive control and molecular grade water was used instead of the sample in the negative control. The microbial community standard was used as per manufacturer’s instructions. All samples including the negative and positive controls were subjected to sequence analysis.

### PCR amplification and DNA sequencing

The PCR amplification of extracted DNA and 16S amplicon sequencing was performed by Novogene Corporation Inc, Sacramento, CA. The V4 region of the 16S ribosomal RNA gene (~ 250 bp) was amplified using the specific primer pair 515F (5′-GTGCCAGCMGCCGCGGTAA-3′) and 806R (5′-GGAC TACHVGGGTWTCTAAT-3′), with the forward primer carrying the barcode sequence. The Phusion^®^ High-Fidelity PCR Master Mix (New England Biolabs) was used to carry out all PCR reactions.

The PCR products were purified by running them on a 2% agarose gel and extracting them using a Qiagen^®^ Gel Extraction Kit. The sequencing library preparation was done using a NEBNext^®^ UltraTM^®^ DNA Library Prep Kit for Illumina. It was quality tested using Qubit 3.0 fluorometer and quantified using Q-PCR. Subsequently, an Illumina HiSeq 2500 platform was utilized for the sequencing of the library, generating ~ 250 bp paired-end raw sequence reads.

### Negative controls

Negative controls were collected from equipment used in SVL collection, laboratory reagents etc. and was subjected to DNA extraction and subsequent sequence analysis. Only one sample for laboratory reagents produced detectable DNA after amplification and was sequenced. Resulting reads were deleted from the subsequent analyses using microDecon^57^, a subtraction tool, prior to statistical analyses.

### Data analysis

The computational analysis of 16S V4 amplicon sequence data was conducted using DADA2 version 1.8, according to the SOP for Illumina data^64^. All DADA2 codes were run using R-Studio version 1.1.4 on R version 3.6.1. Briefly, the quality scores of forward and reverse sequence reads were plotted to identify any drastic drops in sequence quality, specifically towards the ends of the sequences, and no such significant drops were observed. Hence, no specific truncation of the ends of sequences were done during the pre-processing step. However, any sequences that had ambiguous bases and an expected error greater than 2 were removed using the ‘filterAndTrim’ command in DADA2. Subsequently, the error rates for the amplicon sequence data were modeled using ‘learnErrors’function in DADA2 and the sequence reads were then dereplicated using the function derepFastq. The dereplicated sequences were then used for the inference of sequence variants for each sample using the core sample inference algorithms available in DADA2, implemented in the function ‘dada’. Using the mergePairs function, the corresponding forward and reverse inferred sequence variants were then merged to arrive at the full de-noised sequence contigs. The ‘makeSequenceTable’ function was run on these contigs to create the amplicon sequence variant (ASV) table. The ASV table was then subjected to chimera removal and taxonomic classification via removeBimeraDenovo and assignTaxonomy functions. The DADA formatted version of the Silva version 132 reference database^45^ was used as the training dataset for taxonomic classification. All sequences of non-bacterial origin (i.e., chloroplasts, mitochondria, Archaea and Eukaryotes) were removed. The ASV table and the taxonomy file generated by DADA2 was then used for statistical analysis and data visualization, using MicrobiomeAnalyst^22^. Multiple sequence alignment and phylogenetic analysis of representative ASVs were conducted using MEGA7^65^.

### Data filtering and normalization

Low abundance features, defined as those with less than 10 read counts in at least 20% of the samples were removed prior to data normalization. Rarefying of data based on minimum library size, and Total Sum Scaling was performed as data normalization steps to negate the effect of uneven sequencing depth^22^. The resulting filtered and normalized data were used for subsequent statistical analyses and data visualizations.

### Phylogenetic analysis

A total of 104 ASVs along with their taxonomic classifications were used to construct a phylogenetic tree to depict the evolutionary relationships between some of the most abundant bacterial genera found in the current study. ASVs with a relative abundance > 0.05% were selected and when several qualifying ASVs were assigned with the same genus, the representative ASV with the highest relative abundance was chosen. Multiple sequence alignment and phylogenetic tree building (via MUSCLE) was done using algorithms implemented in MEGA7^65^.

### Ethical approval

All methods were performed in accordance with the relevant guidelines and regulations of respective institutions. Animal Use and Care were covered under protocols VM-17-25 and IACUC 20-50 (Oklahoma State University), #17-046 (Louisiana State University), and A18086 (Charles Sturt University).

## Supplementary Information

Supplementary Table S1.

Supplementary Table S2.

Supplementary Table S3.

## Data Availability

The raw sequencing data used in this study is available at the Sequence Read Archive (SRA) of NCBI under the accession number PRJNA784331.
